# Leaky Coupled Waveguide-Plasmon Modes for Enhanced Light–Matter Interaction

**DOI:** 10.3390/s25051550

**Published:** 2025-03-02

**Authors:** Fadi Sakran, Said Mahajna, Atef Shalabney

**Affiliations:** 1 Department of Natural Sciences, Beit Berl College, Beit Berl 4490500, Israel; fadi.sakran@beitberl.ac.il; 2 Physics and Optical Engineering Department, Braude College of Engineering, Karmiel 2161002, Israel; said@braude.ac.il

**Keywords:** leaky coupled waveguide plasmon resonances, sensors, vibro-polariton, vibrational strong coupling

## Abstract

Plasmon waveguide resonances (PWRs) have been widely used to enhance the interaction between light and matter. PWRs have been used for chemical and biological sensing, molecular detection, and boosting other optical phenomena, such as Raman scattering and fluorescence. However, the performances of plasmon-waveguide-based structures have been investigated in the angular interrogation mode, and their potential in different spectral regions has hardly been explored. Moreover, the applications of PWRs have been limited to the weak light–matter coupling regime. In this study, we investigate leaky coupled waveguide plasmon resonances (LCWPRs) and explore their potential to enhance light–matter interaction in different spectral regions. In the weak coupling regime, we demonstrate the potential of LCWPRs for sensing in the near-IR region by detecting heavy water (D_2_O) and ethanol in water. The experimental results show spectral sensitivity of 15.2 nm/% and 1.41 nm/% for ethanol and D_2_O detection, respectively. Additionally, we show that LCWPRs can be used to achieve vibrational strong coupling (VSC) with organic molecules in the mid-IR region. We numerically show that the coupling between LCWPRs and the C=O stretching vibration of hexanal yields a Rabi splitting of 210 cm^−1^, putting the system in the VSC regime. We anticipate that LCWPRs will be a promising platform for enhanced spectroscopy, sensing, and strong coupling.

## 1. Introduction

When electromagnetic radiation is confined within tiny regions of space, the nature of the interaction of the radiation with matter becomes of great interest from both the fundamental point of view and for many optical engineering applications. This radiation confinement, usually accompanied by an extraordinary enhancement of the electric field intensity, accounts for many interesting effects, such as surface-enhanced Raman scattering (SERS), enhanced optical transmission, enhanced absorption, and emission of light, and enables high-resolution microscopy and imaging [1,2,3,4,5]. The outstanding potential of optical modes for sensing and molecular detection, for instance, originates from the volume of interaction of the optical field with the medium to be sensed [6,7,8,9,10]. The combination of the field enhancement and the penetration depth inside the interrogated material determines this interaction volume.

Surface plasmon polaritons (SPPs), collective excitations of the longitudinal charge density on the interface between thin metallic films and their adjacent medium, have been successfully used to enhance electromagnetic fields locally. This enhancement has revealed the ability of extended and localized surface plasmon waves to boost a variety of optical phenomena, such as the absorption and emission of light, surface-enhanced microscopy and spectroscopy, and Raman scattering [7,11,12]. However, surface plasmon waves (SPWs) have a limited penetration depth inside the interrogated medium, ranging from a few hundred nanometers in the visible region to nearly one micron in the near-infrared (NIR) region [13]. These inherent properties have limited the capability of conventional surface plasmon resonance (SPR) sensors to detect low-concentrated materials and large entities such as bacteria and cells. Recently, Bajaj et al. proposed using a plasmonic insulator-metal-insulator (IMI) structure with a higher penetration depth for biofilm detection. The penetration depth of a few microns was achieved with the IMI structure based on the single wavelength of a diverging beam [14].

Conventional optical guided modes generated in planar waveguides and optical fibers have also been used for sensing [15,16,17]. Unlike SPPs that generally suffer from significant dissipation on the metal interface, guided modes have long propagation distances due to their guided nature within the waveguide core. Nevertheless, the moderate field enhancement and the limited penetration depth outside the waveguide region hinder their capabilities for sensing. These inherent properties typically originate from the symmetric environment of these structures [17].

Several configurations in which plasmonic and guided modes are coupled have been proposed, particularly for sensing. In these configurations, metallic and dielectric layers were combined to optimize their performances for sensing and molecular detection, such as the use of long-range surface plasmon resonance (LR-SPR) [18], and metal-insulator-metal, and IMI [19,20]. Coupled waveguide-surface plasmon resonance (CWSPR) or PWR was proposed for the first time by Salamon and Tollin [21,22,23]. In this structure, a dielectric waveguide is coupled to a plasmonic layer (generally made of silver or gold) to generate optical modes in both transverse magnetic (TM) and transverse electric (TE) polarization states. PWRs have proved to be able to monitor mass and structural changes, and have allowed anisotropy information from molecular interactions to be obtained. The main principles, applications, and instrument development of PWRs were recently reviewed by Rascol et al. [24]. Leaky waveguides were also widely used for chemical and biological applications. They can be made of pure dielectric waveguides or by adding a metallic film, the so-called metallic leaky waveguides (MLW) [25]. The state-of-the-art of leaky waveguides for chemical and biological sensing were recently reviewed by Gupta and Goddard [26]. Moreover, the coupling between SPPs and dielectric waveguides may lead to irregular optical phenomena, such as Fano-type resonances with extreme enhancement and confinement of the optical field near the waveguide surface [27]. It is essential to indicate that there are many methods to enhance light–matter interaction in the nanoscale for sensing, such as localized surface plasmon resonance (LSPR), metamaterials, and electromagnetic-induced transparency (EIT) [28,29,30,31,32].

However, most of the coupled waveguide-plasmon configurations were investigated in the visible region, emphasizing the angular behavior where the spectral features were somewhat overlooked [33]. Moreover, the spectral behavior of these modes in the NIR and mid-infrared (MIR) regions has hardly been explored. The increasing interest in these spectral domains is due to the distinct fingerprints of organic molecules and biological species in the IR region, such as molecular vibrations.

Sensing and molecular detection fall into the realm of the weak coupling regime. In this regime, the optical field probes the material without altering its properties. If the interaction strength between the optical field and the interrogated material is sufficiently large, the interaction may enter the strong coupling regime where the basic properties of the material are being modified [34,35,36]. Strong light–matter coupling is achieved by interfacing molecules with a confined electromagnetic field, which is in resonance with a given molecular transition. When a molecular transition is strongly coupled to an optical mode, two new hybrid states, known as polaritons, are formed, separated in energy by the so-called Rabi splitting $\hbar \Omega_{R}$. Strong light–matter coupling has attracted considerable attention in the past few years due to its potential applications in physical and chemical sciences [37]. It has been recently shown that the splitting in the energy levels of molecular components due to strong coupling has great potential to modify the energy landscape of the ground and the excited states. In organic molecules, for instance, this modification directly impacts the material’s fundamental properties, such as altering the work function of organic semiconductors, conductivity, and energy transfer rate [37,38,39,40]. Moreover, the change in the energy landscape of the molecules affects the kinetics of chemical reactions in which the molecule is involved. This has led to unconventional routines for chemical catalysis and bond-selective chemistry [41,42,43,44,45,46,47,48]. 

Recently, VSC has come into focus as a promising physical tool to control molecular properties. The field has expanded considerably since the first experimental evidence of VSC in both solid and liquid states [49,50,51]. It has been shown to alter chemical reactions kinetics, steer reactions selectivity, affect enzyme activity, and modify thermodynamics. Traditionally, polaritons have been engineered using planar Fabry–Perot (FP) cavities to confine the electromagnetic field between two mirrors. To achieve VSC, the molecular vibration should resonate with one of the FP cavity optical modes. As a result, the absorption band of the bare molecule split, and two vibro-polaritons appear instead of the original vibration of the molecule. Reaching considerable coupling strengths requires filling the entire volume of the cavity with the molecular material. In addition, the transition dipole moment of the interacting molecules should be sufficiently large to have a splitting larger than the full-width half maximum (FWHM) of both the molecular transition and the cavity mode. This makes the optical structure crucial in achieving strong coupling in different spectral regions.

In this work, we explore the coupling between extended SPPs and waveguides in the entire dispersion space to generate LCWPRs. We optimize the dispersion of the LCWPR modes in the NIR region for sensing. The dispersion can be carefully tuned to achieve leaky modes with large penetration depths into the sensed medium. Moreover, the intensity of the optical fields of these modes can reach more than two orders of magnitude with respect to the incident field. The co-existence of large penetration depth and enhanced field intensity boosts their interaction volume with the material, thus improving their potential for light–matter interaction applications.

We experimentally examine the potential of the LCWPR modes for sensing by using them to detect D_2_O and ethanol inside pure H_2_O in the NIR region. The experimental results show spectral sensitivity of 15.7 nm/% and 1.41 nm/% for ethanol and D_2_O detection, respectively. In addition, we theoretically study the potential of the LCWPR modes for VSC. We show that, by engineering the dispersion in the MIR region, we can achieve VSC between the LCWPR and molecular vibrations. We tested the strong coupling between LCWPRs and the vibrational transition of the C=O carbonyl bond in hexanal molecules. Our calculations show that the Rabi splitting can reach 210 cm^−1^, at least twice the value reported in the literature for the same transition, using the traditional FP cavities. We show the feasibility of engineering the dispersion of the LCWPR modes to control the interaction strength between the optical field and the interrogated material. Our results show that LCWPRs not only hold promise for sensing and molecular detection but offer an alternative route to strong coupling in an open configuration with the possibility of optically tuning the coupling strength.

## 2. Materials and Methods

### 2.1. Preparation of the Nanophotonic Structure

SF11 glass substrates were cleaned using IPA/ethanol and MilliQ in bath sonication to remove contaminants/dust and dried with a gentle nitrogen blow before use. The metallic film thickness was determined following the optimization in our previous theoretical work [52]. The different layers were custom-designed and optimized according to the desired penetration depth and field intensity. First, we deposited 15 nm silver on the SF11 glass substrate, followed by 5 nm gold. We found that, in single-metal-layer-based SPR structures, silver shows narrower and deeper plasmonic signatures than gold. However, on the other hand, gold is more stable against interaction with other chemicals and materials. Therefore, we chose to have a single silver layer covered by a skinny gold layer for protection. The dispersion of the SF11 prism was taken from [53].

Polymethylmethacrylate (PMMA) was dissolved in toluene (15 wt. %) and mechanically steered at 55 °C for 24 h, cooled to room temperature, and passed through a 0.22 μm nylon filter prior to spin-casting. We used PMMA to generate the waveguide for our study. Following our optimization, spin-casting directly coated the bimetallic silver/gold layer with a 620 nm PMMA layer. The whole structure was attached to an SF11 prism using a refractive index (RI) matching oil. The dispersion of the PMMA in the relevant spectral region was taken from [53].

### 2.2. Optical Set-Up

The whole structure was fixed on a motorized rotational stage with a high resolution (minimal achievable incremental movement ~100 μrad) to enable fine-tuning of the incident angle. The leaky modes were tailored to be in the NIR region. Therefore, a broadband (Thorlabs’ SLS201L) stabilized tungsten-halogen light source with constant intensity in the spectral range from 360 to 2600 nm was used. The polarization of the incident beam (TE or TM) was achieved by an ultra-broadband wire grid polarizer (Thorlabs’ WP25L-UB1) with a minimal extinction ratio of ~800 in the NIR region. The spectrum of the reflected light was collected and analyzed using a NIR spectrometer (AvaSpec-NIR256-2.5-HSC-EVO) with a wavelength range of 1000–2500 nm.

### 2.3. Materials for Sensing Experiments

Heavy water (D_2_O) (#7789200, Sigma-Aldrich, St. Louis, MO, USA) was used as a study case for sensing. The concentration of D_2_O varied by mixing it with pure water suitable for HPLC (#270733, Sigma-Aldrich). The second test material used for sensing is pure ethanol (#64175, Sigma-Aldrich), which was diluted in pure water to vary the concertation and RI. The RIs of the D_2_O and the pure water were modeled according to Kedenburg et al. [54].

### 2.4. Hexanal Absorption for Vibrational Strong Coupling Investigations

To examine the feasibility of the LCWPR for VSC, we used hexanal as a test molecule. To model the RI of hexanal, we measured the absorption spectrum in the MIR region. We used hexanal (#66251, Sigma-Aldrich) as a molecule with prominent and clear absorption bands in the MIR region. The absorption of hexanal was measured using the Attenuated Total Reflection (ATR) module of a standard Fourier Transform IR (FTIR) spectrometer (Nicolet 6700). The pure hexanal was applied on top of the diamond crystal of the ATR accessory and the reflection was acquired in the range of 500–5000 cm^−1^ (wavelength range, 2–20 μm), with a spectral resolution of 4 cm^−1^. The dielectric constant of hexanal was retrieved from the ATR measurement as described in the following section.

### 2.5. Modeling the Dielectric Constant of Hexanal

To analytically calculate the reflectivity and the distribution of the optical field inside the layers, we used an analytic algorithm based on the Abeles matrices [6]. The algorithm enables calculating the reflectivity from a multi-layer structure and the field distribution across the layers.

In order to simulate the optical response of the hexanal in the IR region, the Lorenz model was used to describe molecular polarizability. To fit the IR reflection measurements of the hexanal, the Lorenz model with multiple resonances in the relevant spectral range was assumed as follows [55]:

(1) $$\varepsilon (k)=\varepsilon_{B}-\sum_{j=1}^{N}\frac{f_{j}}{k^{2}-k_{0j}^{2}+ik\Gamma_{mj}}$$

Here, $\varepsilon_{B}{\;,\;f}_{j}\;,\;k_{0j}\;$, and $\Gamma_{mj}\;$ are, respectively, the background dielectric contribution, oscillator strength, resonance wave vector, and the phenomenological damping constant of vibrational band *j*. The absorption intensity of the band is determined by both $f_{j}$ and $\Gamma_{j}\;$, whereas the FWHM is solely governed by $\Gamma_{mj}$. In our fit procedure, all these parameters together were varied to obtain the best fit with the experimental reflection measurement of hexanal. The best fit parameters for the C=O bond absorption band were $f_{j}=3\times 10^{4},k_{0j}=1708\;cm^{-1},\Gamma_{mj}=25\;cm^{-1}\;$ and the background RI is $n_{B}=\sqrt{\varepsilon_{B}}=1.50$.

The dispersion of the metallic layer, on the other hand, was modeled by the Lorenz–Drude model (Equation (2)). In this model, the contributions of the intra-band and the inter-band transitions are explicitly separated, as can be seen in Equation (2) [56]:

$$\varepsilon_{m}=\varepsilon_{free-electrons}+\varepsilon_{bound-electrons}$$

(2) $$\varepsilon_{free-electrons}=1-\frac{f_{0}\omega_{p}^{2}}{\omega (\omega -i\Gamma_{0})};\varepsilon_{bound-electrons}=\sum_{j=1}^{K}\frac{f_{j}\omega_{p}^{2}}{(\omega_{j}^{2}-\omega^{2})+i\omega \Gamma_{j}}$$

In the last two terms, $\omega_{p}$ is the plasma frequency, K is the number of oscillators involved, each with frequency $\omega_{j}$, strength $f_{j}$, and lifetime $1/\Gamma_{j}$.

## 3. Results and Discussion

### 3.1. Investigating LCWPRs for Sensing

**Figure 1:**
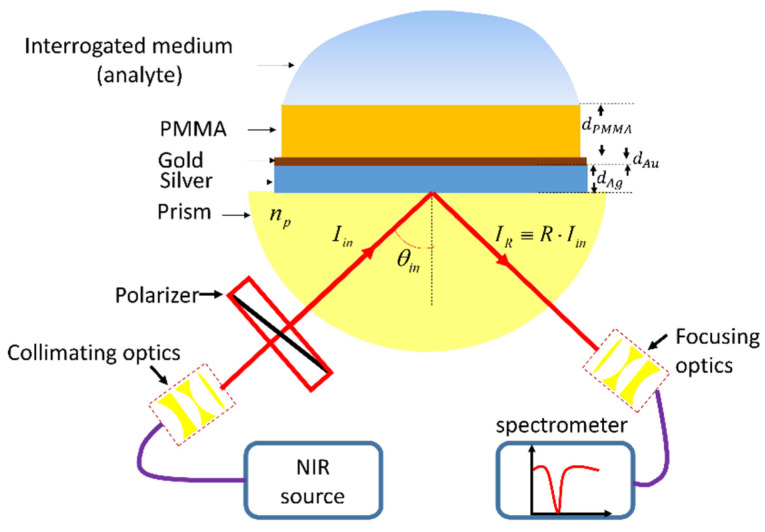
Schematic description of the structure and the optical setup used in this study. SF11 substrate was coated with 15 nm silver layer and 5 nm gold film for protection; then, a 620 nm PMMA layer was spin-coated on top of the gold. The structure was attached to the SF11 prism using RI matching oil (does not appear in the figure). The interrogated medium (analyte) is applied on top of the nanophotonic structure. A broadband thermal source generates the incident beam, and the normalized reflectivity spectrum is recorded in the NIR region.

As described in Section 2, the thin bimetallic layer coated by the PMMA waveguide was used as a platform to excite and investigate the LCWPR for sensing and enhanced light–matter interaction. To probe these modes in both the TM and TE polarization states, we composed the so-called Kretschmann–Raether (KR) configuration. The SF11 substrate on which the bimetallic and PMMA films were coated was attached to an SF11 prism with the aid of RI matching oil. The entire optical setup with the nanophotonic device is schematically described in Figure 1.

In this configuration, one can simultaneously excite the SPPs on the interface between the metal and the PMMA and the guided modes inside the PMMA, as was discussed by Mahajna et al. [52]. Whilst the SPP mode can be excited only in the TM polarization state, the guided modes can be excited in both TE and TM, which enables anisotropic sensing [57]. The capability of probing the anisotropy of the sensed medium was recently reported by Isaacs et al. [19] and addressed in our recent theoretical predictions [52]. Here, we focus on the TE_0_-guided mode inside the PMMA layer and use it to explore the interaction with the interrogated material (analyte) adjacent to the waveguide. In the following, the analyte is applied on top of the nanophotonic structure, and the interaction between the optical mode and the medium is explored by monitoring the reflectivity spectra in the NIR region.

**Figure 2:**
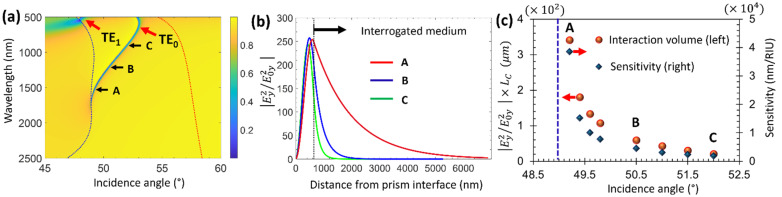
(**a**) Reflectivity color map showing the dispersion of TE_0_ and the onset of TE_1_ modes that can be excited in the structure described in Figure 1. The dashed blue (left side) and red (right side) curves show the critical angle on the water and the PMMA interfaces, respectively. Points A, B, and C indicate three different angles in which the optical field distribution was calculated. (**b**) The normalized intensity of the electric field y-component versus the distance from the prism interface showing the mode structure in three different points (A, B, and C) along the dispersion map. The y-component intensity ($E_{y}^{2}$) is normalized to the incident intensity ($E_{0y}^{2}$). The incidence angles in points A, B, and C are 49.2°, 50.5°, and 52°, respectively. (**c**) The interaction volume of the optical field with the analyte region as described in the text (red solid circles—left axis), and the sensitivity to variations in the analyte RI (blue diamonds—right axis) vs. incidence angle. Points A, B, and C correspond to the same incidence angles that were indicated in (**a**,**b**). The vertical blue dotted line refers to the critical angle at point A (at resonance wavelength 1560 nm).

We first investigate how the penetration depth of the TE_0_ mode varies inside the interrogated medium along the dispersion branch. Figure 2a shows the dispersion of the guided TE_0_ mode and the onset of the TE_1_ mode that appears in the upper part of the dispersion map. The analyte in these calculations is water, and its dispersion was considered in the entire NIR region [54]. The guided nature of the TE_0_ mode dramatically evolves when moving along the dispersion map. In Figure 2b, we show the normalized intensity of the electric field at three different angles along the TE_0_ dispersion branch. While the mode has a genuine guided nature in points B and C, it is leaky in the vicinity of the total reflection line, which is indicated by the left-side dashed blue line in the dispersion map. This leakage increases the interaction volume of the mode with the interrogated medium, as shown in point A, by one and two orders of magnitude compared to points C and B, respectively (Figure 2b). Moreover, Figure 2b shows that the normalized intensity of the optical field at the waveguide interface is enhanced by more than two orders of magnitude. In the context of using evanescent fields for sensing, it was shown that the sensitivity is governed by the overlap integral of the optical field inside the sensed medium [6,58]. This overlap integral is proportional to the product between the penetration depth and the field intensity at the interface. Therefore, due to the extended penetration depth and the large field intensity, the LCWPR should have prominent potential for bio-sensing. Figure 2c shows the correlation between the structure’s sensitivity and its interaction volume with the optical field. Here, sensitivity is defined as the shift in the resonance wavelength per RI unit (RIU). Given that the optical field inside the analyte has an evanescent character, the intensity can be written as $I\left(z\right)=I_{0}e^{\frac{\left(z-z_{0}\right)}{L_{c}}}$, where $I_{0}$ is the normalized field intensity at the PMMA/analyte interface ($z_{0}$) and $L_{C}$ is the characteristic length at which the intensity drops to $\frac{1}{e}I_{0}$ inside the analyte. The interaction volume of the optical field with the analyte is proportional to $I_{0}\times L_{C}$. In Figure 2c, we plot this product for different incidence angles. The correlation is clearly observed between the interaction volume and the sensitivity of the structure to variations in the analyte RI.

First, we investigate the potential of the enhanced interaction volume of the LCWPR for sensing and molecular detection. In one study case, we used the TE_0_ mode to detect D_2_O in pure water. We have chosen this case due to the importance of D_2_O detection and monitoring in many medical and industrial applications. D_2_O is toxic at high concentrations, so knowing its safe level in biological systems using a miniaturized device should be very helpful for in vivo bioassays. In addition, detecting D_2_O is essential for treating chronic infections and crucial in monitoring environmental contamination [59,60,61,62,63]. So far, complicated or bulky methods have been used to detect D_2_O, such as SERS or FTIR based techniques [59,61].

**Figure 3:**
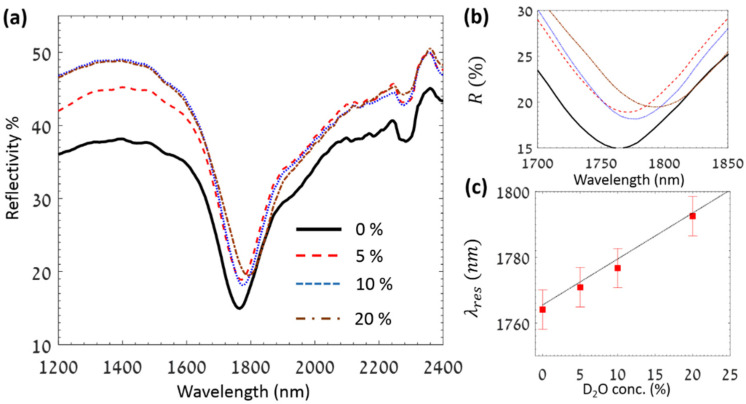
(**a**) Reflectivity spectrum for different concentrations of D_2_O in pure water. (**b**) Magnified illustration of the dip region showing the evolution in the resonance wavelength for 0%, 5%, 10%, and 20% D_2_O in water. (**c**) Resonance wavelength versus D_2_O concentration showing the linear dependence for low concentration values.

To examine the LCWPR for D_2_O detection, the incidence angle inside the prism was first tuned near the critical curve of the pure water, as was depicted by Figure 2a. Then, a mixture of H_2_O–D_2_O with varying concentrations of the D_2_O replaced the pure water, and the shift in the resonance wavelength was monitored. Figure 3a,b show the evolution in the reflectivity spectrum and the resonance wavelengths, respectively, for different concentrations of D_2_O. Considering the dispersion of H_2_O and D_2_O, the measured sensitivity is $4\times 10^{4}\;nm/RIU$, corresponding to a shift of 1.416 nm/% of D_2_O in H_2_O. Recent work showed that 0.25% of heavy water in a D_2_O–H_2_O mixture could be detected in the MIR region [64]. In this report, the detection mechanism relies on the evolution in the differential absorption of D_2_O around a 4 µm wavelength using a tuned laser in the MIR region. Additional recent works have addressed the detection of D_2_O in water either by optical or chemical approaches [65,66]. These methods mostly require sophisticated fabrication techniques to generate the optical structure for sensing or using specific luminescent probes. Using our configuration, on the other hand, one can reach comparable detection limits in the NIR region without any need for complicated fabrication methods.

**Figure 4:**
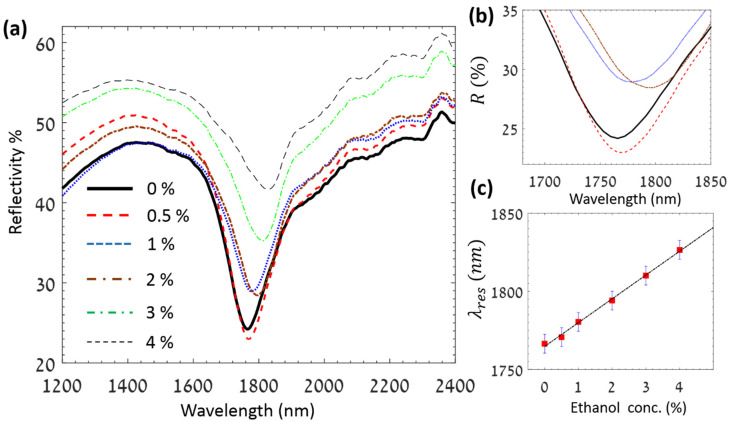
(**a**) Reflectivity spectrum for different concentrations of ethanol in pure water. (**b**) Magnified illustration of the dip region showing the evolution in the resonance wavelength for 0%, 0.5%, 1%, 2% ethanol in water. (**c**) Resonance wavelength versus ethanol concentration showing the linear dependence for low concentration values.

In another case, we examined the potential of the LCWPR for the detection of ethanol in pure water. Low concentrations of ethanol in pure water were prepared and tested using our platform. The evolution in the reflectivity spectrum versus the change in the ethanol concentration is shown in Figure 4. Here, the observed relative shift was 15.2 nm/% of ethanol in water, revealing at least one order of magnitude enhancement in the detection sensitivity with respect to other recent techniques [15,20].

### 3.2. Investigating the Potential of LCWPRs for Vibrational Strong Coupling

In the former two cases, we have explored the effect of the large interaction volume of the LCWPR with matter in the weak coupling regime. In this regime, the optical mode probes the medium without altering the material’s properties. In the strong coupling regime, on the other hand, an optical mode can be resonantly coupled to the absorption band of a given material, forming two new hybrid polaritonic states [37].

Resonance interaction between a molecular transition and a confined electromagnetic field can reach the strong coupling regime where a coherent exchange of energy between light and matter becomes reversible. In this case, two new hybrid states separated in energy are formed instead of independent eigenstates. The conditions for the observation of Rabi splitting ($\hbar \Omega_{R})$ obtained from a linear dispersion model or cavity quantum electrodynamics (CQED) are the following. The Rabi frequency must be larger than the damping rate of both the optical field and the molecular transition [34,35,37].

The strong coupling is typically achieved by locating the target material inside an FP cavity (acting as an optical resonator) where the molecules interact with the optical modes of the resonator. The strong coupling between the molecular transition and the cavity mode results in a splitting in the molecular absorption band. The key parameters in determining the splitting amount are the number of molecules within the optical mode volume and the coupling strength between the molecules and the optical field [37].

**Figure 5:**
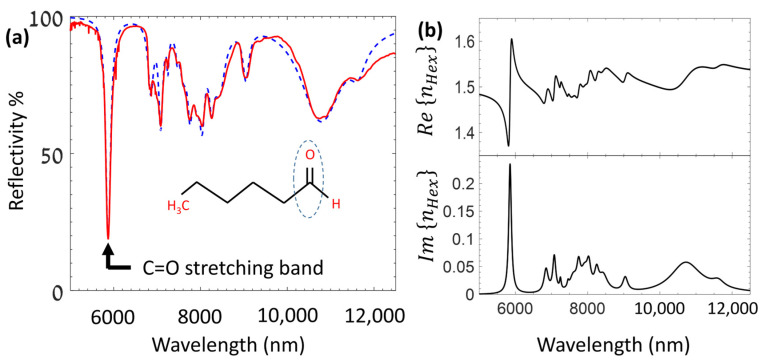
(**a**) ATR spectrum of hexanal (red solid line) and the best fit according to the dielectric constant retrieved by the Lorenz model (blue dashed line). A strong absorption band of the C=O stretching vibration is observed at 5.872 μm (1702 cm^−1^) with FWHM of 0.14 μm (40 cm^−1^). The inset shows the molecular structure of hexanal and the carbonyl group. (**b**) The real (top panel) and imaginary (bottom panel) parts of the hexanal RI as was retrieved from the best fit (using the Lorenz model) with the measured spectrum.

To assess the potential of the LCWPR in achieving strong coupling with molecular vibrations in the IR region, we studied the interaction between the leaky modes and hexanal as a test molecule. Hexanal is liquid at room temperature and was processed in neat form. We first measured the absorption spectrum of the hexanal in the IR region using the ATR accessory in the FTIR spectrometer. A small amount of pure hexanal was applied to the ATR crystal (made of diamond), and the reflectivity spectrum was recorded. This experiment enabled us to calculate the dielectric function of hexanal in the IR region. We applied the Lorenz model, described in Section 2, to retrieve the whole manifold of the absorption bands of hexanal in the MIR region. Figure 5a shows the measured ATR spectrum of pure hexanal, showing a strong absorption band centered at 5.872 μm ($1703\;{cm}^{-1}$) with FWHM of 40.5 cm^−1^. This strong absorption band is assigned to the C=O stretching mode of hexanal and will be the molecular vibration of interest in this study. The other absorption bands characteristic of hexanal’s C–H bending vibrations are fairly separated from the C=O band that we intend to target in this study [66]. Figure 5a also shows the theoretical fit of the reflectivity using the predicted dielectric constant according to the Lorenz model, which gives the best fit with the measured spectrum. The hexanal RI’s real and imaginary parts, as predicted by the Lorenz model, are shown in Figure 5b.

**Figure 6:**
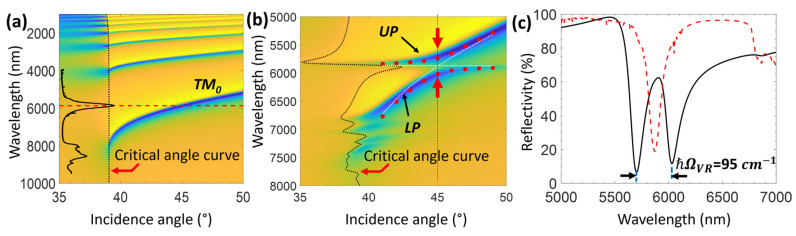
(**a**) Dispersion map of the TM modes of the structures used to study VSC between the C=O band and the LCWPR. The structure comprises a 10 nm Ag film and a 2030 nm ZnSe layer. The absorption spectrum of the hexanal is superimposed (for illustration) on the dispersion map to show the C=O band dip around 5872 nm (1702 cm^−1^), depicted by the horizontal red dashed line. (**b**) Dispersion of the TM_0_ mode with the hexanal showing the anti-crossing around the absorption band of the C=O vibration and the formation of two new hybrid states: the UP and LP. The dotted white curves show the dispersion of the uncoupled system: the TM_0_ mode and the bare molecular absorption band. The red dots show the position of the UP and LP according to the coupled harmonic oscillator model. (**c**) Cross-section of the TM_0_ dispersion, showing the splitting in the C=O absorption band. The value of the normal Rabi splitting is $\hbar \Omega_{VR}=95\;{cm}^{-1}$. The red dashed line describes the absorption spectrum of the bare hexanal molecule.

Since we investigate the coupling between the LCWPR and the C=O vibration in the MIR, the prism and the waveguide material should be compatible with this spectral region. Here, we use ZnSe as a waveguide instead of PMMA and the diamond prism instead of SF11. The structure that we propose to study VSC between hexanal and the LCWPR consists of a thin Ag film coated with a ZnSe waveguide. This structure is attached to a diamond prism, and the hexanal is applied on top of the ZnSe waveguide. The thickness of the ZnSe layer determines the number of the allowed guided modes and their distribution across the spectral region, whereas the silver film thickness influences the depth and width of the optical modes. The RIs of the ZnSe layer and the diamond prism were considered according to the MIR spectral region [53,67]. Figure 6a shows the manifold of TM modes that can be excited in our structure, using a 10 nm Ag film and a 2030 nm thick ZnSe waveguide. The structure was designed to tune the coupling between the TM_0_ mode and the C=O band at an incidence angle of 45°. An anti-crossing in the dispersion map is obtained when adding the hexanal on top of the ZnSe waveguide (Figure 6b). A genuine signature of the strong coupling is the formation of two new polaritonic states due to the coupling between the bare molecule and the optical field. Figure 6b clearly shows the formation of the upper polariton (UP) and the lower polariton (LP) states when the dispersion splits into two branches near the absorption band. When these polaritons are a mixture of a molecular vibration and an optical mode, they have a vibro-polariton character. This vibro-polariton is a superposition of the bare localized molecular state and the delocalized optical mode.

Figure 6c shows a cross-section of the dispersion map at an incidence angle of 45°, showing a clear normal mode splitting of the vibrational absorption band of C=O vibration when placed within the leaky mode. This splitting indicates a strong coupling between the LCWPR and the carbonyl group of hexanal. The resulting formation of vibro-polaritons gives a Rabi splitting of 95 cm^−1^, which is larger than both the FWHM of the bare molecular vibration (40 cm^−1^) and the leaky mode (92 cm^−1^), constituting further evidence that our system is indeed in the strong coupling regime.

In addition to using the transfer matrix approach to calculate the reflectivity of the coupled system, we examined the coupled harmonic oscillator model to predict the positions of the UP and LP following the strong coupling between the molecular vibration and the LCWPR [68,69,70]. We modeled the optical mode and the vibrational band as linear harmonic oscillators with a given eigenfrequency and damping rate. The coupling strength was deduced from the calculated Rabi splitting in the dispersion map. The eigenfrequencies of the UP and LP can be associated with dressed states, that is, the oscillator frequencies of the optical mode and the vibrational band in the presence of mutual coupling [69,70]. These eigenfrequencies are presented as red dots on the dispersion map in Figure 6b.

To investigate the effect of the interaction volume between the hexanal and the LCWPR on the VSC strength, we varied the waveguide thickness to control the incidence angle at which the carbonyl C=O band couples with the optical mode. We examined the coupling strength at three incidence angles (42°, 41°, and 39.5°) near the critical angle curve. To obtain VSC at these angles (42°, 41°, and 39.5°), we tuned the ZnSe waveguide thickness to 1840 nm, 1755 nm, and 1590 nm, respectively. One can see that the anti-crossing in the dispersion maps and the splitting in the reflectivity spectrum increase when the absorption band of the molecule couples with the optical mode near the critical angle boundary. The Rabi splitting obtained at 42°, 41°, and 39.5° is 123 cm^−1^, 144 cm^−1^, and 210 cm^−1^, respectively. The splitting behavior versus the incidence angle indicates that the LCWPR offers a platform for VSC with the possibility to control the coupling strength. Recently, Hertzog et al. reported on a splitting of 101 cm^−1^ for the C=O stretching band of hexanal when it is coupled to a FP cavity with plasmonic arrays [71]. Here, we see a significant improvement in the coupling strength due to the enhanced interaction volume between the LCWPR and the coupled molecules.

**Figure 7:**
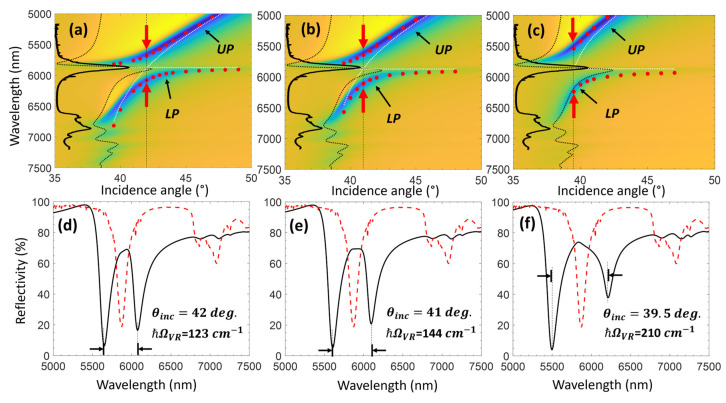
To obtain VSC between the LCWPR and the C=O vibration at different angles, the thickness of the ZnSe was varied. We tuned the ZnSe waveguide thickness to 1840 nm, 1755 nm, and 1590 nm to obtain VSC at incidence angles of 42°, 41°, and 39.5°, respectively. (**a**–**c**) Show the dispersion of the TM_0_ modes for the three thicknesses: 1840 nm, 1760 nm, and 1590 nm, respectively. The black dotted curve shows the distortion in the critical angle near the C=O absorption band. The horizontal red line depicts the C=O absorption band with the bare molecule absorption spectrum superimposed on the dispersion (only for illustration). The dotted white curves show the dispersion of the uncoupled LCWPR and the molecular band. The red dots on the dispersion maps show the position of the UP and LP according to the coupled oscillator model. The vertical red arrays indicate the Rabi splitting when the LCWPR and the molecular band are in resonance (zero detuning). (**d**–**f**) Show the reflectivity spectrum for the incidence angles 42°, 41°, and 39.5°, respectively, showing the evolution in the Rabi splitting when coupling between the vibrational band and the LCWPR approaches the critical angle curve.

The dispersion maps in Figure 7 show a distortion in the critical angle curve near the absorption band of the coupled molecule. This distortion (depicted by the dotted black curves on the dispersion maps) results from the strong anomaly of the hexanal RI near the C=O absorption band, which enables the leaky modes to move further toward smaller angles, as can be seen from the dispersion of the lower and upper vibro-polariton states.

**Figure 8:**
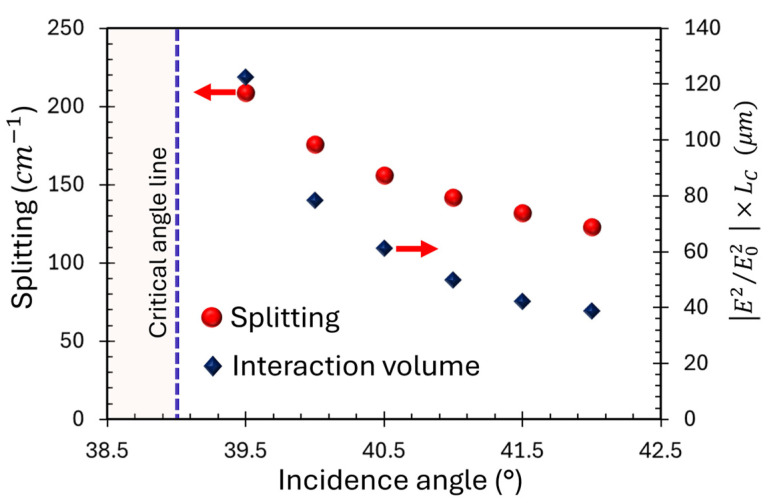
The interaction volume of the optical field with the analyte region as described in the text (blue diamonds—right axis), and the Rabi splitting (red solid circles—left axis) vs. incidence angle. The dashed vertical blue line indicates the critical angle of the structure.

To gain insight into the origin of the enhanced splitting, we calculated the interaction volume of the LCWPR with the molecular medium in different incidence angles. The waveguide thickness varied to tune the TM_0_ mode in resonance with the molecular absorption. Figure 8 shows the interaction volume of the optical field with the hexanal medium and the Rabi splitting versus the incidence angle. The apparent correlation between the interaction volume and the Rabi splitting confirms the role of the leaky character of the LCWPR in enhancing the interaction strength and consequently boosting the coupling between the optical mode and the molecular transition.

## 4. Conclusions

The potential of LCWPRs for enhancing light–mater interaction was presented. The dispersion of LCWPRs can be tailored in different spectral regions to enhance the interaction volume between the optical field and the interrogated material. The optical fields’ large penetration depth and high intensity boost the interaction with the matter under study. It has been experimentally shown that LCWPRs can be used for sensing and molecular detection in the NIR region. Monitoring D_2_O and ethanol in water was presented as a study case for sensing in the NIR region with lower detection limits of 15.2 nm/% and 1.41 nm/% for ethanol and D_2_O, respectively.

The potential of LCWPRs for strong light–matter coupling was also explored. In the strong coupling regime, the interaction between a given molecular transition and the optical field alters the material properties. The coupling between LCWPRs, tuned to the MIR region, and hexanal’s carbonyl vibrational transition (C=O stretching band) was numerically investigated. Our results show that the LCWPR modes can be tailored to achieve VSC with the C=O stretching band with normal Rabi splitting of 210 cm^−1^ (12% of the molecular vibration energy). This splitting is larger than the optical mode and the molecular vibration spectral widths, which unambiguously puts the system in the VSC regime. The coupling strength between the optical field and the transition under strong coupling can be controlled by tailoring the dispersion curve of the LCWPR modes. These results offer the LCWPR as a promising platform to explore the potential of polaritonic chemistry and the ultra-strong coupling regime. In addition, LCWPRs have the potential for sensing and molecular detection in the NIR and MIR regions with improved sensitivity.

## Data Availability

The original contributions presented in this study are included in the article. Further inquiries can be directed to the corresponding author.
